# Metachronous triple primary malignancies: a case report of NGS-guided multidisciplinary management and literature review

**DOI:** 10.3389/fonc.2026.1705144

**Published:** 2026-02-19

**Authors:** Xiangxue Li, Lihua Zhang, Xiaowei Wang, Jing Lv, Caiqi Liu, Siyi Zhang, Xiaoxuan Li, Jialin Song, Wensheng Qiu, Shufen Zhao

**Affiliations:** 1 Department of Oncology, Key Laboratory of Cancer Molecular and Translational Research, The Affiliated Hospital of Qingdao University, Qingdao University, Qingdao, China; 2 Department of Gastroenterology, The Affiliated Hospital of Qingdao University, Qingdao University, Qingdao, China

**Keywords:** case report, multidisciplinary team, multiple primary malignancies, next-generation sequencing, metachronous triple primary malignancies

## Abstract

**Rationale:**

Although the incidence of multiple primary malignancies (MPM) is increasing, triple primary malignancies (TPM) remain extremely rare. The pathogenesis of MPM is currently unclear, and there is a lack of optimal management strategies. Herein, we report a case of metachronous TPM involving both the respiratory tract and the digestive tract.

**Objective:**

This report aims to analyze the clinical characteristics of the patient and emphasize the significant role of next-generation sequencing (NGS) and multidisciplinary team (MDT) in the management of MPM.

**Diagnoses and interventions:**

A 72-year-old male patient was diagnosed with a lung cancer and underwent surgical resection. Two years later, he was diagnosed with synchronous gastric cancer, rectal cancer, and bilateral pulmonary metastases. Guided by NGS and multiple MDT consultations, the patient received four cycles of individualized neoadjuvant chemotherapy combined with targeted therapy, followed by radical resection and postoperative adjuvant chemotherapy alongside targeted treatment. Currently, the patient’s pulmonary metastatic lesions are being managed with ongoing targeted therapy.

**Outcomes:**

The patient currently maintains a good quality of life. During the follow-up period, no recurrence or metastasis of gastric or rectal cancer was observed, and the bilateral lung metastases showed a sustained partial response.

**Conclusions:**

The management of TPM is considerable complex. MDT guided by NGS can be instrumental in formulating optimal, personalized treatment strategies for such patients, which may contribute to improved clinical outcomes and quality of life.

## 1 Introduction

Cancer remains a major global health challenge, with incidence and mortality rates showing a steady upward trend worldwide (1, 2). According to 2020 global cancer statistics, approximately 19.3 million new cancer cases and nearly 10 million cancer-related deaths were reported, with projections suggesting a 47% increase in new cases by 2040 (3).

Multiple primary malignancies (MPM), defined as the occurrence of two or more histologically distinct primary tumors in a single individual (4), have a reported prevalence ranging from 2.4% to 8% (5). Based on the time interval between the occurrence of the first and second primary tumor, the International Agency for Research on Cancer/International Association of Cancer Registries classified MPM into two types: synchronous MPM referred to tumors that occur simultaneously or within 6 months after the first primary malignant tumor, while metachronous MPM was defined as tumors that develop with an interval of more than 6 months (6, 7). Advances in cancer screening technologies and improved patient survival rates have led to an increased detection rate of MPM. However, most cases of MPM involve dual primary cancers, whereas triple primary malignancies (TPM) are exceptionally rare, accounting for only 0.03%–0.16% of all cases (8). The pathogenesis of MPM was currently considered to be a multifactorial process. Aging, genetic susceptibility, environmental carcinogen exposure, and immune deficiency were all important driving factors (9, 10). Concurrently, the role of metabolic diseases such as obesity was increasingly being recognized (11). They might create a favorable microenvironment for the occurrence of MPM by inducing chronic inflammation and insulin resistance, etc. Moreover, previous radiotherapy or chemotherapy treatments were also regarded as potential iatrogenic risk factors, although the exact causal relationships required further clarification. Although cases of MPM have been reported in the literature (**Supplementary Table 1**), clinical management remains challenging due to therapeutic conflicts arising from tumor heterogeneity, difficulties in identifying metastatic origins, and the cumulative risks of multi-organ toxicity.

Therefore, we report a rare case of metachronous triple primary malignancies involving both the respiratory and digestive systems. Through next-generation sequencing analysis and multidisciplinary team (MDT) collaboration, we investigate the molecular characteristics, therapeutic strategies, and clinical implications of the case.

## 2 Case presentation

### 2.1 Patient information

A 72-year-old male, a former smoker with a 30 pack-year history (discontinued prior to admission), presented to our hospital on September 14, 2022, with a one-month history of cough and sputum. The patient denied alcohol consumption and any family history of malignancy.

### 2.2 Initial diagnosis and management

**Figure 1:**
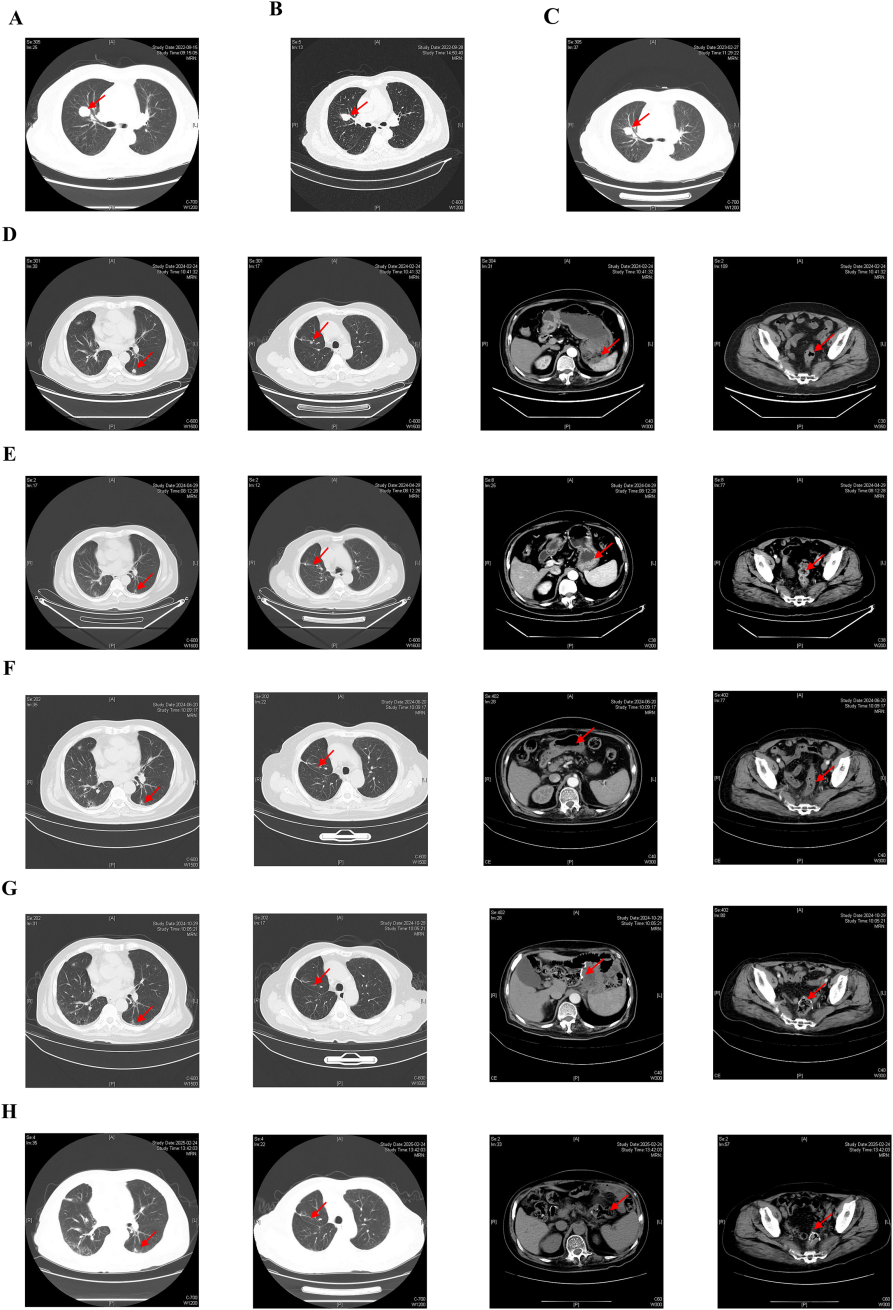
CT scans at different time points. **(A)** Chest CT scan of lung cancer baseline. **(B)** Chest CT scan after symptomatic treatment with ceftazidime plus methylprednisolone. **(C)** Chest CT scan showing progression after follow-up five months. **(D)** Chest CT scan showing pulmonary metastases progression after lung cancer surgey and abdominal CT scan of gastric cancer and rectum cancer baseline. **(E)** Chest and abdominal CT scans showing PR after 2 cycles treatment of almonertinib and XELOX. **(F)** Chest and abdominal CT scans showing sustained PR after 4 cycles treatment of almonertinib and XELOX. **(G, H)** Chest and abdominal CT scans showing pulmonary metastases sustained PR and no recurrence or metastasis of the gastrointestinal tumors after 4 cycles of almonertinib and XELOX following with surgey and follow-up in February 2024, respectively. CT, computed tomography; PR, partial response; XELOX, oxaliplatin plus capecitabine.

**Figure 2:**
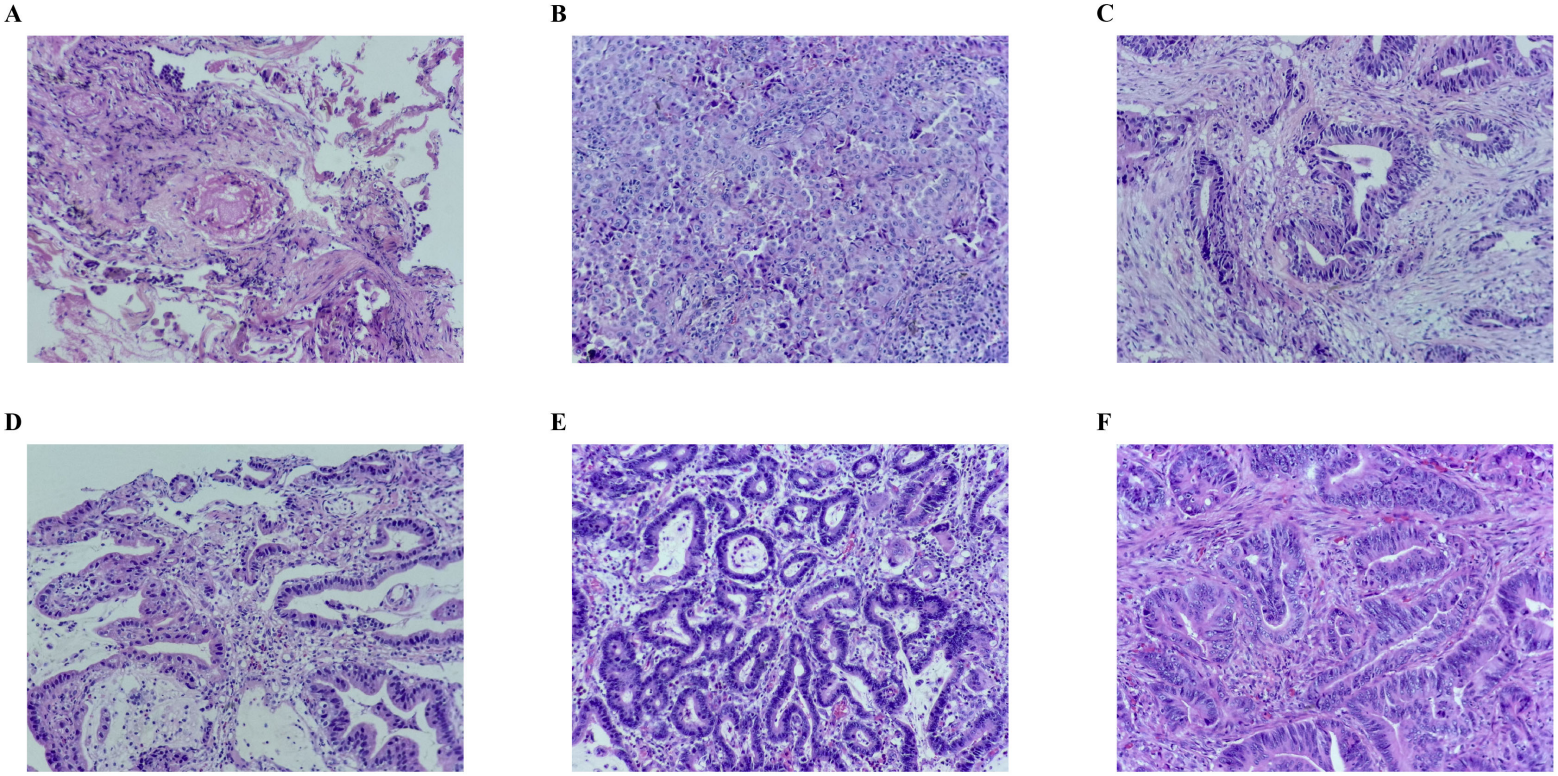
HE staining of the tumor at different time points. All pictures were taken at a 200-fold magnification using a light microscope. **(A)** Biopsy specimen of EBUS-GS-TBLB revealed no malignant tumor cells. **(B)** Specimen after lung surgey revealed infiltrating adenocarcinoma. **(C)** Biopsy specimen of gastric cancer after 4 cycles treatment revealed no exclusion of canceration. **(D)** Biopsy specimen of rectum cancer after 4 cycles treatment revealed adenocarcinoma. **(E)** Specimen after gastric cancer surgey revealed gastric antral adenocarcinoma. **(F)** Specimen after rectum cancer surgey revealed raised-type adenocarcinoma. HE, hematoxylin and eosin; EBUS-GS-TBLB, endobronchial ultrasound-guided sheath-guided transbronchial lung biopsy.

The chest computed tomography (CT) at the local hospital revealed a right upper lobe mass (2.5 × 2.5 cm) with bilateral pulmonary nodules. Contrast-enhanced chest CT in our hospital showed lobulation, spiculation, and pleural indentation in the upper lobe of the right lung and several roundish small nodules in bilateral lungs (**Figure 1A**). Endobronchial ultrasound-guided transbronchial lung biopsy with guide sheath (EBUS-GS-TBLB) performed on September 15, 2022, biopsy pathology showed no evidence of malignancy (**Figure 2A**). After empirical anti-infective therapy with ceftazidime plus methylprednisolone, a follow-up CT of the patient indicated no improvement (**Figure 1B**). Subsequent antimicrobial therapy (levofloxacin plus fluconazole) and repeat EBUS-GS-TBLB similarly yielded negative results.

After five months, follow-up enhanced CT displayed the lung mass was larger than before (**Figure 1C**). Video-assisted thoracoscopic surgery with right upper lobectomy and middle lobe wedge resection on March 13, 2023 was performed, postoperative pathology confirmed primary lung adenocarcinomas: a solid-pattern invasive adenocarcinoma in the right upper lobe and an acinar-pattern invasive adenocarcinoma in the middle lobe, with bronchial lymph node metastasis (2/5 nodes positive) (**Figure 2B**). Immunohistochemical staining revealed TTF-1(+), NapsinA (+), and PD-L1 tumor proportion score <1% (**Table 1**). Genetic testing revealed no driver gene mutations. We organized the initial MDT discussion: The bilateral pulmonary nodules measured 2-5 mm and were radiologically considered likely benign. Meanwhile, they were too small to allow for technically feasible and safe percutaneous lung biopsy for a clear pathological nature. Based on the postoperative pathological stage of pT2aN1, negative driver gene, and low PD-L1 expression, adjuvant platinum-based doublet chemotherapy was recommended for the patient in accordance with the Chinese Society of Clinical Oncology (CSCO) guidelines. Concurrently, regular follow-up was advised to monitor the minute nodules and clarify their nature. However, the patient ultimately declined the proposed chemotherapy.

**Table 1:** The immunohistochemical profile of lung tissue.

TTF-1	NapsinA	p40	CD50	ALK(D5F3)N	PD-L1 TPS	Ki-67
+	+	–	–	–	<1%	+,10%

TPS, tumor proportion score; +, positive; -, negative.

### 2.3 Second diagnosis and management

In February 2024, the patient was re-admitted for a 10-day history of diarrhea. External gastroscopy and colonoscopy identified (1) an irregular gastric antral mucosal protrusion (moderately-to-poorly differentiated adenocarcinoma) and (2) a circumferential rectal mass (moderately differentiated adenocarcinoma) occupying one-third of the lumen. CT demonstrated enlarged bilateral pulmonary nodules, as well as possible gastric cancer and rectal cancer (**Figure 1D**). We performed comprehensive genetic testing on the postoperative lung specimen as well as the endoscopic biopsy specimens from the stomach and rectum based on the same standardized gene detection platform (GeneSeeq). NGS profiling of all lesions revealed distinct molecular signatures: (1) EGFR L858R mutation (38.7% allele frequency) in lung lesion, (2) NRAS Q61K mutation (22.1% allele frequency) in rectal lesion, and (3) no driver mutations in gastric lesion (**Table 2**). We organized the MDT again to guide further treatment: The pulmonary, gastric, and rectal lesions respectively represented distinct primary malignancies, with bilateral pulmonary metastases likely originating from the primary lung adenocarcinoma, considering the negative result of surrounding lymph nodes in abdominopelvic CT. Since the gastric and rectal cancers could not be resected endoscopically and the presence of multiple pulmonary metastases, surgical treatment was not advised at this stage. Therefore, treatment was recommended to targeted therapy (almonertinib 110 mg qd) for the EGFR L858R-mutant lung cancer, along with neoadjuvant chemotherapy (XELOX regimen: oxaliplatin 130 mg/m² + capecitabine 1000 mg/m²) for the gastrointestinal cancers.

**Table 2:** Overview of this patient’s next-generation sequencing results.

Gene name	Mutations	Mutation frequency*		
		Rectum	Stomach	Lung
EGFR	p.L858R exon 21 missense mutation			15.43%
NRAS	p. G12D exon 2 missense mutation	46.06%		
ATM	c.8418 + 2T>C intron 57 shear mutation		19.64%	
TP53	p. R175H exon 5 missense mutation	36.88%		
TSC1	p. k148Mfs*21 exon 6 frameshift mutation	45.72%		
BAP1	c.68-2A>C intron 2 scissor mutation		24.62%	
CBLB	p.R608* exon 12 nonsense mutation	26.86%		
CREBBP	c.1893_1941 + 168del exon 9 shear mutation		4.81%	
CTNNB1	c.74_241 + 56del exon 3 shear mutation	37.56%		
CTNNB1	p. S33Y exon 3 missense mutation			0.79%
GATA6	p. k500Q fs*14 exon 5 frameshift mutation			1.10%
NOTCH2	p.R2400* exon 34 nonsense mutation			
TET2	p.R544* exon 3 nonsense mutation	17.52%		
ARID1B	p. G720E exon 5 missense mutation		0.53%	
ATM	p.N2875S exon 59 missense mutation		16.83%	
BRCA2	p. P655L exon 11 missense mutation		3.55%	
CEBPA	p. N283S exon 1 missense mutation			17.03%
EPHA3	p. D238Y exon 3 missense mutation		1.30%	
ERBB3	p. E548K exon 14 missense mutation	1.36%		
ERBB3	p. D691N exon 18 missense mutation		3.52%	
ETV1	p. G243A exon 9 missense mutation		1.53%	
GNAS	p. R26C exon 1 missense mutation		1.52%	
GRINA2	p. P140L exon 4 missense mutation		0.82%	
MSH2	p. Ll28v exon 3 missense mutation		1.89%	
PAK3	p.E226* exon 10 nonsense mutation		11.45%	
POLE	p. P441L exon 13 missense mutation		10.77%	
ROS1	p. V2054G exon 39 missense mutation		1.57%	
TEK	p.clll8y exon 23 missense mutation		2.26%	
TSHR	p. D410N exon 10 missense mutation	0.56%		

*The results were based on the GeneSeeq testing platform.

### 2.4 Treatment and follow-up

**Figure 3:**
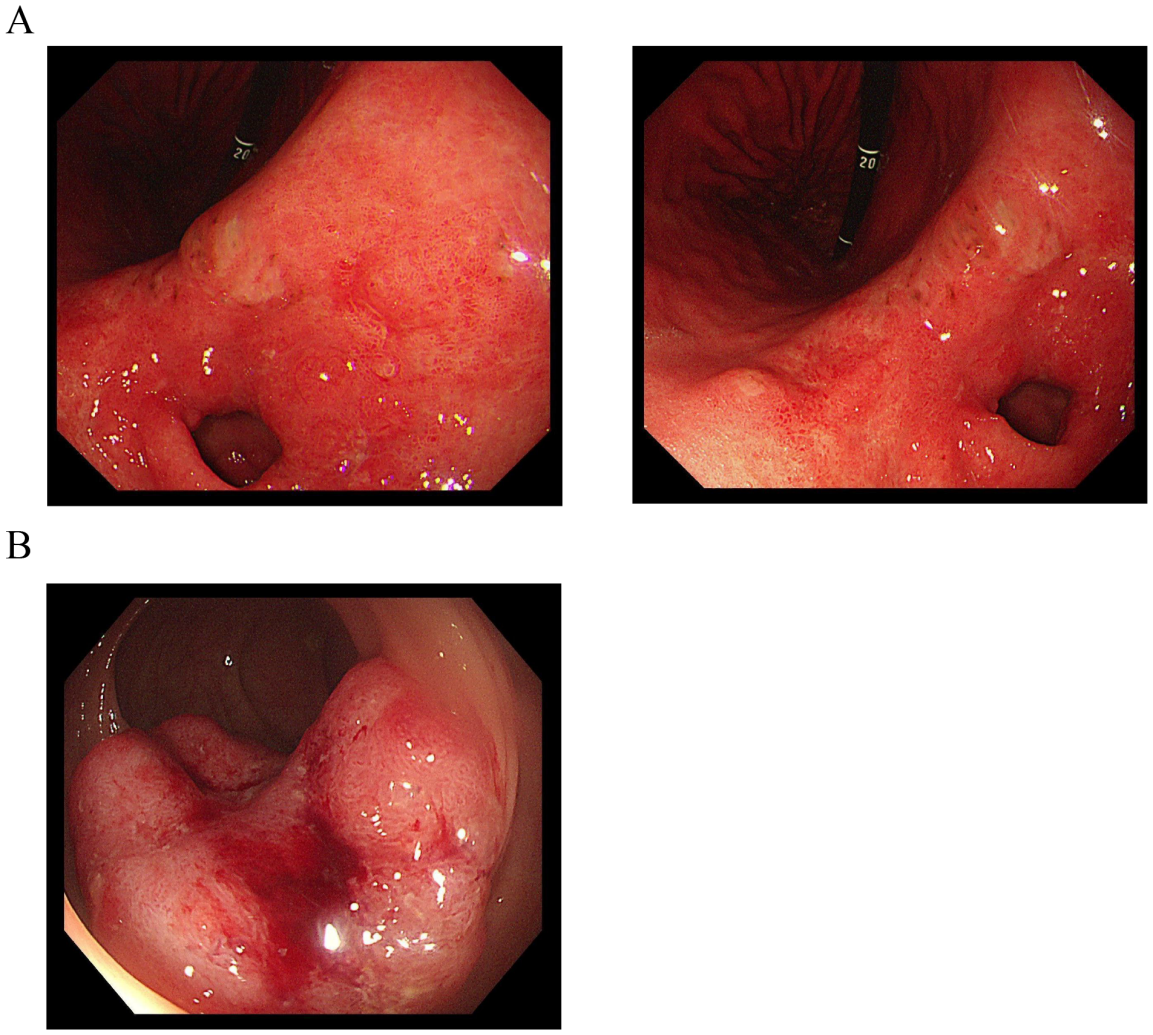
Endoscopy of stomach and rectum after 4 cycles of treatment. **(A)** Gastroscopy revealed rough and raised mucosa in the gastric antrum, with redness, congestion, and poor peristalsis. **(B)** Colonoscopy revealed an irregular ulcer encircling half of the rectal lumen, located 13-17 cm from the anal verge.

**Figure 4:**
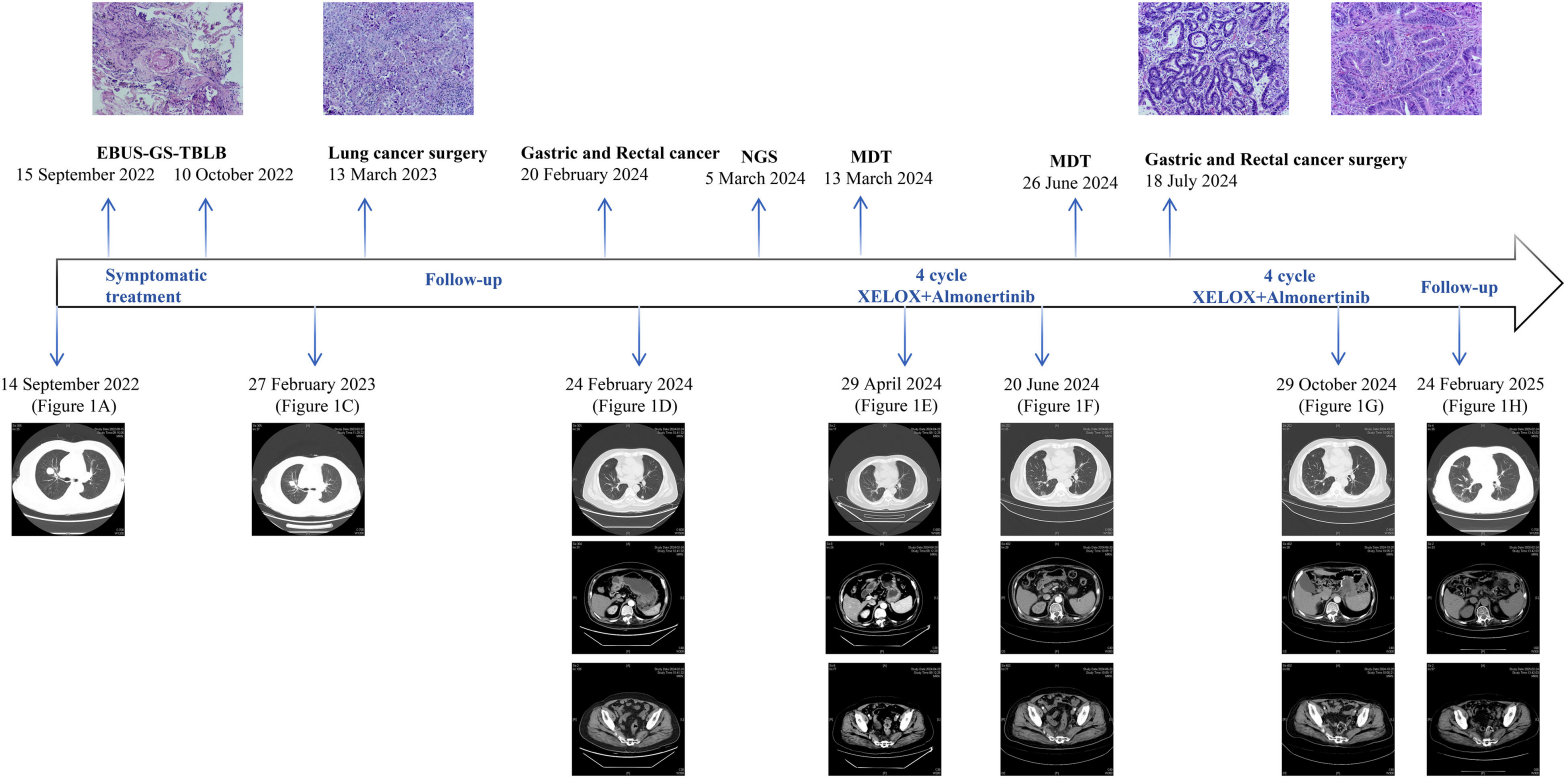
The flowchart of treatment process a patient with triple primary malignant.

After two and four cycles, CT evaluation showed partial response (PR) in lung metastases, with a trend toward shrinkage of lesions in the stomach and rectum (**Figures 1E, F**). Repeat endoscopy in June 2024 revealed rough and raised mucosa in the gastric antrum and an irregular ulcer located 13-17 cm from the anal verge (**Figures 3A, B**). Biopsy pathology showed gastric high-grade dysplasia and rectal ulcerative adenocarcinoma (**Figure 2C, D**). Based on the evaluation of the patient’s CT scans and gastrointestinal endoscopic results, the primary gastric and rectal lesions showed significant improvement following neoadjuvant therapy. Concurrently, targeted therapy effectively controlled the pulmonary metastases, and the patient’s physical condition was favorable. To maximize the guarantee of the patient’s long-term quality of life and survival benefit, radical surgical resection for the gastric and rectal tumors was recommended according to a comprehensive MDT proposal. In July 2024, laparoscopic radical gastrectomy and colectomy were performed. Postoperative pathology confirmed gastric adenocarcinoma (pT4aN0, TRG 2) and rectal adenocarcinoma (pT2N0, TRG 3), both microsatellite stable (MSS) (**Figures 2E, F**). Postoperative NGS testing of 196 tumor genes for rectal lesion revealed NRAS mutation. The patient subsequently completed four cycles of adjuvant XELOX chemotherapy and almonertinib targeted therapy. The patient is currently undergoing targeted therapy for the bilateral pulmonary metastases and has regular follow-up CT examinations, sustained PR in lung metastases with no evidence of gastrointestinal recurrence (**Figure 1G, H**). The flowchart of the treatment process is shown in **Figure 4**.

## 3 Discussion

A comprehensive and systematic literature search was conducted in the PubMed database to identify case reports of triple and more primary malignancies published between 2000 and 2025 (**Supplementary Table 1**). The search was performed using the following combination of keywords: (“multiple primary malignancies” OR “triple primary malignancies” OR “quadruple primary malignancies” OR “quintuple primary malignancies”) AND “case report”. MPM was classified into synchronous and metachronous types using a six-month threshold (12). We found that synchronous cases accounted for 65% of MPM instances, while metachronous cases comprised 35%, which was largely consistent with a previous retrospective study finding (7). The increase in the incidence of metachronous MPM might be related to the increase in average lifespan and the availability of more effective anti-tumor treatment options (6).

The etiology of MPM remains unclear and may involve various pathogenic mechanisms such as genetic predisposition, environmental exposures, and treatment-related factors. Some hereditary syndromes significantly increased the risk of MPM. For example, Lynch syndrome (LS) was closely associated with colorectal cancer, endometrial cancer and other tumors (13). It was reported that the risk of developing colorectal cancer in patients with LS ranged from 30% to 74% (14). LS was characterized by germline mutations in DNA mismatch repair (MMR) genes. Defects in MMR function led to microsatellite instability and the inability to correct DNA replication errors, thereby promoting carcinogenesis (15). Additionally, mutations in BRCA genes, which played a critical role in DNA repair, significantly increased the risk of breast and ovarian cancer (16, 17). Furthermore, antitumor therapy might serve as a contributing factor to the development of second primary malignancies. Chemotherapy drugs, such as alkylating agents, could damage the DNA of normal cells while killing tumor cells, leading to new mutations or even carcinogenesis. A case-control study found that the relative risk of leukemia in breast cancer and ovarian cancer patients treated with cyclophosphamide significantly increased (18). Similarly, although radiotherapy was targeted to specific areas, it could still affect surrounding normal tissues and potentially induce malignancies. However, studies suggested that this risk remained relatively narrow (19, 20). Moreover, shared carcinogenic exposures could also lead to the occurrence of MPM, such as smoking, alcohol consumption, and obesity. Smoking was a clear and significant risk factor for various types of cancer, with systemic detrimental effects. Substantial evidence indicated that tobacco use was strongly associated with a significantly increased incidence of malignancies such as lung cancer, head and neck squamous cell carcinoma, and bladder cancer (21–24). Meanwhile, previous observational studies have confirmed that smokers faced a significantly elevated risk of developing MPM compared to never-smokers (25, 26). Carcinogens in tobacco could directly or indirectly damage DNA, in addition, it could induce oxidative stress and chronic inflammatory responses (27). The patient is an elderly male with a long-term history of smoking, which might be a significant contributing factor to his development of MPM. Furthermore, as shown in the supplementary table1, 58% of the patients had a chronic smoking history, which might suggest the role of both smoking and aging in the pathogenesis of MPM.

Notably, NGS can play a vital role in guiding cancer treatment, contributing to personalized precision medicine and may improve prognosis for patients. A study conducted by the University of Michigan Rogel Cancer Center involving over 1,000 advanced cancer patients reported the following results (28): Among 132 patients receiving sequencing-guided therapy, 37.1% achieved clinical benefit, with 19.7% attaining exceptional response (defined as disease control for at least one year). Furthermore, some studies have found that tumor patients receiving genomic guided therapy experienced significantly longer progression-free survival (PFS) (29–31). In this case, the lung cancer target identified by subsequent genetic testing provided a crucial prerequisite for the patient to undergo radical surgery for gastrointestinal tumors and achieve survival benefits. This highlighted the indispensability of dynamic genetic monitoring in identifying potential beneficiaries of targeted therapy. However, it must be recognized that while targeted therapy can bring significant disease relief, acquired resistance is widespread (32, 33), and achieving long-term durable remission remains a major challenge. Future advances may depend on the continuous development of precision medicine and the exploration of optimal drug combination strategies. Additionally, genomic profiling can be instrumental in elucidating clonal origin, particularly for metachronous MPM, thereby facilitating the critical differential diagnosis between an independent primary tumor and a recurrence or metastasis (34, 35). A study has shown for patients with cancer of unknown primary origin, NGS helped resolve tissue-of-origin identification in nearly half of cases (50.9%), providing clinicians with enhanced insights to inform the selection of effective standard therapies and targeted treatments (28). However, NGS results were substantially affected by sample preparation, processing, and the amount of DNA input (36). Therefore, standardized NGS assays with uniform gene panels, sequencing depth, and bioinformatics pipelines were crucial for accurate diagnosis. In this case, the patient underwent NGS testing on specimens from three organs in the same institution. Mutant genes that were not detected in the previous test were found in the lung specimens, providing strong evidence for a clear diagnosis and the formulation of subsequent treatment plans. Notably, the discrepancy in the detection results was primarily attributed to the technical limitations of the initial test, leading to a false-negative outcome. The second genetic test employed sequencing with greater depth and a broader gene panel, which significantly enhanced detection sensitivity. Additionally, the two tests might sample different regions of the tumor tissue, which also suggested the potential possible of intratumor genetic heterogeneity (37). At the same time, it also emphasized the importance of homogenized management of genetic testing.

There was currently no established consensus regarding the optimal management of MPM, particularly for metachronous MPM, which continued to present substantial clinical challenges. Given MPM potential involvement of multiple organs and systems, the complexity of these diseases made it difficult for any single discipline to formulate an optimal management strategy. Not only should we take into account the conflicts and priorities in different tumor treatment decisions, but also consider issues such as the patient’s tolerance and subsequent quality of life. The MDT approach is a widely endorsed treatment model, particularly for cancer patients, and has been established as an internationally recognized standard in the management of malignant tumors (38). The study by Freeman et al. reported that patients managed through an MDT approach were more likely to receive standardized therapy earlier (39). Furthermore, a systematic review encompassing 27 studies also suggested that cancer patients presented at the MDT were more likely to undergo more accurate and complete staging, as well as receive more appropriate oncological management (40). It was worth noting that MDT might also have an impact on the survival outcome of patients. A study has shown that the median overall survival and PFS for lung cancer patients treated through MDT were increased by 8 months and 5 months respectively (41). Similarly, many studies have found that MDT might have a positive correlation with the survival rate of patients (42, 43). In this specific case, we conducted three MDT discussions for this patient at different treatment stages, formulating the individualized best treatment plan for him. The treatment strategy emphasized multidisciplinary coordination: EGFR-targeted therapy (almonertinib) combined with chemotherapy achieved PR in lung metastases, while delayed radical resection of gastrointestinal cancers minimized perioperative metastatic risks. This “systemic control first, localized intervention second” strategy illustrates how sequential MDT interventions can aid in balancing therapeutic efficacy and safety (44).

Despite these potential benefits, over 90% of MPM cases currently lacked standardized NGS testing (45–47), leading to continued reliance on suboptimal therapies. Of the 80 cases reviewed, only 18 underwent genetic testing, although not every primary lesion was sequenced. In addition, merely 12 cases received MDT management. Unfortunately, only 5% of cases involved the combined application of NGS and MDT in the diagnostic and therapeutic process. Therefore, an NGS-guided MDT approach could be considered to help to determine clonal origins and track resistance patterns. This TPM case highlighted the potential value of integrating molecular profiling with multidisciplinary strategies to improve outcomes for this rare, high-risk population. At the same time, it must be recognized that although the MDT model is widely recommended globally, its full implementation still faces practical obstacles such as medical resource allocation and clinical workload costs. In the management of MPM, the core leading role of the clinical oncologist is particularly crucial. Therefore, a more flexible oncotherapy team (OTT) model can serve as a potential and pragmatic supplement to the MDT. The OTT can not only consolidate the initial MDT decision but may also deputize it in the multiple line settings of the entire management process (48).

Research indicated that lung cancer had the highest incidence among male patients with metachronous first primary cancer (49). Compared to the general population, lung cancer patients exhibited a significantly elevated risk of developing MPM, with reported incidence rates ranging from 13.4% to 22% (50, 51). A retrospective study found that among MPM cases involving lung cancer, colorectal cancer, esophageal cancer, and thyroid cancer were the most common accompanying metachronous malignant tumors (52). Another study similarly suggested that colorectal cancer was one of the common second primary tumors in lung cancer survivors (25). Furthermore, previous study has found that colorectal cancer and lung cancer, as well as gastric cancer and lung cancer, ranked among the top five common combinations of metachronous MPM (43). Collectively, these epidemiological observations raise the possibility of an association between a primary lung cancer diagnosis and an elevated risk of subsequent gastrointestinal malignancies, particularly colorectal cancer.

However, it is also crucial to recognize the limitations of this case report. Firstly, despite the absence of a family history of tumors in this patient, germline genetic testing was not performed, making it impossible to completely rule out the potential impact of a hereditary tumor syndrome. Additionally, based on the management strategies and experiences of a single case, the observed results cannot lead to general conclusions, and their broader applicability requires validation in larger-scale future studies. Finally, although this case was reported as an example of successful management, the existence of selection bias cannot be excluded.

This case represented the first reported occurrence of metachronous dual primary gastrointestinal malignancies following lung cancer, while demonstrating the potential survival advantage of mutation-directed targeted therapy in advanced disease. The MDT management model guided by NGS may provide a novel reference for the management of similar cases in the future.

## 4 Conclusion

This case illustrated the complexity of MPM management, where molecular profiling of multiple lesions helped clarify tumor origins and guided personalized strategies through multidisciplinary collaboration. Our approach combining standardized genetic testing with stepwise therapeutic may contribute to long-term survival in this advanced case. While the experience from this single case cannot establish general principles, it underscores the potential value of combining MDT with precision medicine in the management of complex MPM. Standardized genetic testing may serve as the foundation for treatment strategies in such complex scenarios.
