# Supplementary material for: Metachronous triple primary malignancies: a case report of NGS-guided multidisciplinary management and literature review
Source: Front Oncol. 2026 Feb 19;16:1705144. doi: 10.3389/fonc.2026.1705144 (PMC12961439; doi:10.3389/fonc.2026.1705144)
Supplement: Supplementary file 1 [file DataSheet1.doc]

Supplement Table 1: Summary of cases of multiple primary malignancies with more than two primary location (2000 to 2025).

| Case  Number | Report  Year | Age  (years) | Sex | Country | Smoking  Status | Drinking  Status | Family history of malignancy | MPM  Location | MPM  Classification | MDT | NGS | | Reference |
| --- | --- | --- | --- | --- | --- | --- | --- | --- | --- | --- | --- | --- | --- |
|  |  |  |  |  |  |  |  |  |  |  | Tissue | Co-  mutated Gene |  |
| 1 | 2001 | 18 | Female | Hungary | Never | Never | NM | Retina;  Maxillofacial region;  Maxillofacial region | Metachronous | No | - | - | [1] |
| 2 | 2003 | 73 | Male | Japan | NM | NM | NM | Gallbladder;  Common bile duct;  Pancreas | Synchronous | No | - | - | [2] |
| 3 | 2003 | 66 | Male | Japan | NM | NM | NM | Lymph gland;  Lung | Metachronous | No | - | - | [3] |
| 4 | 2003 | 74 | Male | USA | Smoker | Never | No | Bladder;  Kidney;  Prostate;  Urethra | Synchronous | No | - | - | [4] |
| 5 | 2004 | 56 | Female | Japan | Never | Drinker | No | Breast;  Colon;  Brain | Metachronous | No | - | - | [5] |
| 6 | 2008 | 73 | Male | Japan | NM | NM | No | Stomach;  Esophagus;  Jejunum | Metachronous | No | - | - | [6] |
| 7 | 2011 | 78 | Female | Morocco | NM | NM | NM | Skin;  Cervix uteri;  Uncertain | Metachronous | No | - | - | [7] |
| 8 | 2011 | 59 | Female | Jordan | NM | NM | NM | Uterus;  Cecum;  Kidney;  Ovary;  Breast;  Lung;  Oviduct;  Nasopharynx | Metachronous | No | - | - | [8] |
| 9 | 2013 | 57 | Male | Korea | NM | NM | NM | Colon;  Liver;  Spine | Metachronous | No | - | - | [9] |
| 10 | 2013 | 56 | Female | Turkey | NM | NM | NM | Skin;  Lung;  Breast | Synchronous | No | - | - | [10] |
| 11 | 2013 | 33 | Male | China | NM | NM | No | Colon;  Appendix;  Liver | Synchronous | No | - | - | [11] |
| 12 | 2013 | 71 | Male | Iran | Never | NM | NM | Kidneys; Prostate;  Breast | Metachronous | No | - | - | [12] |
| 13 | 2013 | 79 | Male | China | NM | NM | No | Colon and Rectum | Synchronous | No | - | - | [13] |
| 14 | 2013 | 77 | Male | Japan | Smoker | NM | No | Pancreas;  Stomach;  Cecum | Synchronous | No | Pancreas  Stomach;  Cecum | No | [14] |
| 15 | 2013 | 67 | Female | India | NM | NM | No | Small intestine;  Breast;  Ovary | Metachronous | No | - | - | [15] |
| 16 | 2014 | 67 | Male | Japan | Smoker | NM | NM | Bladder;  Prostate;  Penis | Synchronous | No | - | - | [16] |
| 17 | 2014 | 66 | Male | Greece | Smoker | NM | No | Cecum;  Lung;  Kidney | Synchronous | No | - | - | [17] |
| 18 | 2014 | 64 | Female | Greece | Smoker | NM | NM | Breast;  Oviduct;  Uterus | Synchronous | No | - | - | [17] |
| 19 | 2015 | 51 | Male | China | NM | NM | No | Colon | Synchronous | No | - | - | [18] |
| 20 | 2015 | 66 | Male | Italy | NM | NM | NM | Lung;  Colon;  Oropharynx;  Prostate | Synchronous | Yes | - | - | [19] |
| 21 | 2015 | 57 | Female | USA | Never | Drinker | Yes | Skin;  Breast;  Lymph gland | Synchronous | Yes | - | - | [20] |
| 22 | 2016 | 63 | Female | China | Neve | Neve | No | Esophagus;  Lung;  Thymus | Synchronous | No | - | - | [21] |
| 23 | 2016 | 49 | Male | China | NM | NM | NM | Stomach;  Colon;  Rectum | Synchronous | No | - | - | [22] |
| 24 | 2016 | 60 | Female | USA | NM | NM | Yes | Colon;  Kidney;  Endometrium | Synchronous | Yes | Colon;  Uncertain | Uncertain | [23] |
| 25 | 2016 | 42 | Female | Japan | NM | NM | Yes | Colon and Rectum | Synchronous | No | - | - | [24] |
| 26 | 2016 | 72 | Female | USA | Smoker | NM | Yes | Lung | Synchronous | No | Right  Lung | No | [25] |
| 27 | 2017 | 60 | Male | Japan | NM | NM | NM | Bile duct | Metachronous | No | - | - | [26] |
| 28 | 2017 | 56 | Male | Korea | NM | NM | NM | Stomach | Synchronous | No | - | - | [27] |
| 29 | 2017 | 56 | Male | China | NM | NM | NM | Lung;  Liver;  Kidney | Metachronous | No | - | - | [28] |
| 30 | 2017 | 78 | Male | Romania | NM | NM | Yes | Prostate;  Bladder;  Kidney | Synchronous | No | - | - | [29] |
| 31 | 2018 | 66 | Female | China | NM | NM | NM | Appendix | Synchronous | No | - | - | [30] |
| 32 | 2018 | 77 | Female | Japan | Never | NM | NM | Lung | Synchronous | No | Lung | No | [31] |
| 33 | 2018 | 43 | Male | Brazil | Smoker | Drinker | NM | Esophagus | Synchronous | No | - | - | [32] |
| 34 | 2018 | 63 | Female | China | NM | NM | NM | Uterus;  Cervix;  Fallopian tube | Synchronous | No | - | - | [33] |
| 35 | 2018 | 61 | Male | USA | Never | NM | No | Kidney | Synchronous | No | - | - | [34] |
| 36 | 2018 | 55 | Male | China | NM | NM | NM | Colon;  Kidney  Duodenum | Synchronous | Yes | Uncertain | - | [35] |
| 37 | 2018 | 32 | Female | China | Never | NM | Yes | Lung;  Kidney;  Thyroid | Synchronous | Yes | Lung;  Kidney;  Thyroid | No | [36] |
| 38 | 2019 | 81 | Male | China | Never | Drinker | NM | Lung;  Prostate;  Bladder | Synchronous | No | Lung | - | [37] |
| 39 | 2019 | 42 | Male | China | NM | NM | NM | Colon;  Bladder;  Lung | Metachronous | No | - | - | [38] |
| 40 | 2019 | 79 | Male | Japan | Smoker | Drinker | Yes | Stomach;  Colon;  Prostate;  Bladder;  Skin;  Pancreas | Metachronous | No | - | - | [39] |
| 41 | 2019 | 58 | Male | China | NM | NM | NM | Colon;  Thyroid;  Kidney | Metachronous | No | - | - | [40] |
| 42 | 2019 | 63 | Female | Sri Lanka | NM | NM | NM | Rectum;  Breast; Kidney | Metachronous | Yes | - | - | [41] |
| 43 | 2019 | 56 | Female | China | Never | Never | No | Cervix; Endometrium;  Ovary; Stomach | Synchronous | Yes | - | - | [42] |
| 44 | 2019 | 51 | Male | Thailand | Smoker | NM | NM | Pancreas;  Lymph gland;  Hypopharynx | Metachronous | No | - | - | [43] |
| 45 | 2020 |  | Female | China | NM | NM | NM | Uterus;  Colon;  Breast | Metachronous | No | - | - | [44] |
| 46 | 2020 | 57 | Female | Philippines | Never | Never | Yes | Right nasolabial;  Thyroid;  Pituitarium | Synchronous | No | - | - | [45] |
| 47 | 2020 | 51 | Female | China | NM | NM | Yes | Breast;  Uterus;  Ovary;  Fallopian tube | Metachronous | No | - | - | [46] |
| 48 | 2020 | 63 | Male | Saudi Arabia | Smoker | NM | No | Stomach;  Colon;  Kidney | Synchronous | No | - | - | [47] |
| 49 | 2020 | 66 | Male | China | Smoker | NM | No | Esophagus;  Lung;  Liver | Synchronous | No | Esophagus;  Lung;  Liver | No | [48] |
| 50 | 2020 | 57 | Male | Egypt | NM | NM | NM | Blood;  Lymph gland;  Liver | Metachronous | No | - | - | [49] |
| 51 | 2020 | 83 | Male | Japan | Smoker | Drinker | NM | Colon;  Skin;  Oral;  Stomach | Metachronous | No | - | - | [50] |
| 52 | 2021 | 70 | Male | China | NM | NM | No | Esophagus;  Stomach;  Colon | Metachronous | No | Esophagus;  Stomach;  Colon | TP53 | [51] |
| 53 | 2021 | 71 | Male | Saudi Arabia | NM | NM | No | Stomach;  Pancreas;  Rectum | Synchronous | Yes | - | - | [52] |
| 54 | 2021 | 67 | Female | USA | Never | NM | Yes | Skin;  Lymph gland;  Leg | Metachronous | No | Leg | - | [53] |
| 55 | 2021 | 72 | Male | USA | Smoker | NM | NM | Thyroid;  Lung | Synchronous | No | - | - | [54] |
| 56 | 2021 | 50 | Male | China | Smoker | Drinker | Yes | Esophagus;  Stomach;  Jejunum | Synchronous | No | - | - | [55] |
| 57 | 2021 | 30 | Female | China | NM | NM | No | Breast;  Brain;  Liver | Metachronous | No | - | - | [56] |
| 58 | 2022 | 61 | Male | China | NM | NM | Yes | Bladder;  Lymph gland;  Lung | Synchronous | Yes | - | - | [57] |
| 59 | 2022 | 59 | Male | China | NM | NM | NM | Lymph gland;  Rectum;  Liver | Synchronous | Yes | - | - | [58] |
| 60 | 2022 | 55 | Female | Iran | NM | NM | No | Rectum;  Lymph gland;  Groin | Synchronous | No | - | - | [59] |
| 61 | 2022 | 79 | Female | USA | Smoker | NM | No | Blood;  Skin;  Lung;  Kidney | Metachronous | No | - | - | [60] |
| 62 | 2022 | 77 | Female | China | NM | NM | No | Esophagogastric Junction;  Duodenum;  Pancreas | Synchronous | Yes | Esophag-ogastric Junction | - | [61] |
| 63 | 2022 | 64 | Male | China | Smoker | Drinker | NM | Bladder;  Prostate;  Lung | Synchronous | No | Lung | - | [62] |
| 64 | 2023 | 63 | Female | China | NM | NM | NM | Stomach;  Colon;  Rectum | Synchronous | No | - | - | [63] |
| 65 | 2023 | 50 | Male | China | Smoker | NM | Yes | Bladder;  Thyroid;  Prostate | Metachronous | No | - | - | [64] |
| 66 | 2023 | 57 | Male | Spain | Smoker | NM | NM | Bladder;  Kidney;  Prostate | Synchronous | No | - | - | [65] |
| 67 | 2023 | 59 | Male | China | NM | NM | No | Nasopharynx;  Thyroid;  Kidney | Synchronous | No | Nasopharynx  Thyroid | BRAF | [66] |
| 68 | 2023 | 72 | Male | China | NM | NM | NM | Testis;  Stomach;  Bladder;  Breast | Metachronous | No | Uncertain | - | [67] |
| 69 | 2023 | 79 | Female | China | NM | NM | No | Finger;  Bladder;  Toe;  Liver | Metachronous | No | Serum | KRAS | [68] |
| 70 | 2023 | 63 | Female | Vietnam | NM | NM | No | Thyroid;  Colon;  Kidney;  Bladder | Metachronous | No | - | - | [69] |
| 71 | 2023 | 66 | Female | Canada | Smoker | Never | Yes | Lung;  Breast | Synchronous | Yes | Left  Lung | - | [70] |
| 72 | 2024 | 55 | Male | China | Smoker | Drinker | NM | Esophagus;  Oropharynx;  Oral | Metachronous | No | - | - | [71] |
| 73 | 2024 | 69 | Male | Vietnam | NM | NM | NM | Skin;  Lung;  Thyroid | NM | Yes | Uncertain | - | [72] |
| 74 | 2024 | 60 | Female | USA | Smoker | NM | Yes | Lung | Metachronous | Yes | - | - | [73] |
| 75 | 2024 | 50 | Female | China | NM | NM | NM | Uterus;  Ovary;  Breast | Metachronous | No | Uterus;  Ovary | BRCA2;  PALB2;  RECQL4;TP53 | [74] |
| 76 | 2024 | 44 | Male | China | NM | NM | Yes | Lung;  Blood | Metachronous | No | Skin | - | [75] |
| 77 | 2024 | 71 | Male | China | Smoker | Drinker | No | Lung;  Bile duct;  Prostate | Synchronous | No | Lung;  Bile duct;  Prostate | No | [76] |
| 78 | 2024 | 67 | Female | Romania | Smoker | NM | No | Skin;  Cervix;  Colon;  Bladder | Metachronous | No | - | - | [77] |
| 79 | 2024 | 44 | Female | Iran | Never | Never | Yes | Breast;  Stomach;  Thyroid | Metachronous | No | - | - | [78] |
| 80 | 2025 | 69 | Male | China | Smoker | Drinker | NM | Esophagus;  Stomach;  Colon | Synchronous | No | - | - | [79] |

MPM, Multiple primary malignancies; MDT, ‌Multi-Disciplinary Treatment‌; NGS, ‌Next-Generation Sequencing‌; NM, No mentioned,

**References:**

1. Márta U, Zsuzsanna S, József B, Zsolt N, Béla S, György S. Rare incidence of three consecutive primary tumors in the maxillofacial region: retinoblastoma, leiomyosarcoma, and choriocarcinoma: case report. *J Craniofac Surg.* 2001;12(5):464-468.

2. Sato K, Maekawa T, Yabuki K, et al. A case of triple synchronous cancers occurring in the gallbladder, common bile duct, and pancreas. *J Gastroenterol.* 2003;38(1):97-100.

3. Tokuchi Y, Kamachi M, Harada M, et al. Synchronous triple lung cancers after treatment for non-Hodgkin's lymphoma: metachronous quadruple cancers. *Intern Med.* 2003;42(10):1031-1034.

4. Demandante CG, Troyer DA, Miles TP. Multiple primary malignant neoplasms: case report and a comprehensive review of the literature. *Am J Clin Oncol.* 2003;26(1):79-83.

5. Kitayama T, Marubayashi S, Hayamizu K, et al. Allochronic overlapping malignancies after renal transplantation in a patient with p53 gene mutation: report of a case. *Surg Today.* 2004;34(5):473-476.

6. Kato Y, Tsuyuki A, Kikuchi K, et al. Primary jejunal adenocarcinoma as part of multiple primary cancers of the digestive tract. *J Gastroenterol Hepatol.* 2008;23(4):673-677.

7. Mesmoudi M, Boutayeb S, Mahfoud T, et al. Triple malignancy in a single patient including a cervical carcinoma, a basal cell carcinoma of the skin and a neuroendocrine carcinoma from an unknown primary site: A case report and review of the literature. *J Med Case Rep.* 2011;5:462.

8. Slem A, Abu-Hijlih R, Abdelrahman F, et al. Eight primary malignancies: case report and review of literature. *Hematol Oncol Stem Cell Ther.* 2011;4(4):185-187.

9. Jung YS, Kim SH, Ha SK, Kim SD, Lim DJ. Triple primary origin tumor: a case report. *Korean J Spine.* 2013;10(2):91-93.

10. Kurul S, Akgun Z, Saglam EK, Basaran M, Yucel S, Tuzlali S. Successful treatment of triple primary tumor. *Int J Surg Case Rep.* 2013;4(11):1013-1016.

11. Guoliang S, Dongsheng H. Triple synchronous malignant tumors of colon, appendix and liver: A case report with literature review. *Pak J Med Sci.* 2013;29(1):237-238.

12. Zargar-Shoshtari MA, Saffari H, Moslemi MK. Metachronous occurrence of triple malignancies of kidneys, prostate, and breast. A case report and review of the literature. *Case Rep Urol.* 2013;2013:194620.

13. Yeh CC, Hsi SC, Chuu CP, Kao YH. Synchronous triple carcinoma of the colon and rectum. *World J Surg Oncol.* 2013;11:66.

14. Ohtsubo K, Ishikawa D, Nanjo S, et al. Synchronous triple cancers of the pancreas, stomach, and cecum treated with S-1 followed by pancrelipase treatment of pancreatic exocrine insufficiency. *JOP.* 2013;14(5):515-520.

15. Takalkar U, Asegaonkar BN, Kodlikeri P, Asegaonkar S, Sharma B, Advani SH. An elderly woman with triple primary metachronous malignancy: A case report and review of literature. *Int J Surg Case Rep.* 2013;4(7):593-596.

16. Ogawa S, Yasui T, Taguchi K, Umemoto Y, Kojima Y, Kohri K. The probability of involvement of human papillomavirus in the carcinogenesis of bladder small cell carcinoma, prostatic ductal adenocarcinoma, and penile squamous cell carcinoma: a case report. *BMC Res Notes.* 2014;7:909.

17. Sakellakis M, Peroukides S, Iconomou G, Boumpoucheropoulos S, Kalofonos H. Multiple primary malignancies: a report of two cases. *Chin J Cancer Res.* 2014;26(2):215-218.

18. Cheng J, Liu X, Shuai X, Deng M, Gao J, Tao K. Synchronous triple colorectal carcinoma: a case report and review of literature. *Int J Clin Exp Pathol.* 2015;8(8):9706-9711.

19. Testori A, Cioffi U, De Simone M, et al. Multiple primary synchronous malignant tumors. *BMC Res Notes.* 2015;8:730.

20. Williamson CW, Paravati A, Ghassemi M, et al. Five Simultaneous Primary Tumors in a Single Patient: A Case Report and Review of the Literature. *Case Rep Oncol.* 2015;8(3):432-438.

21. Song X, Shen H, Li J, Wang F. Minimally invasive resection of synchronous triple primary tumors of the esophagus, lung, and thymus: A case report. *Int J Surg Case Rep.* 2016;29:59-62.

22. Yang L, Zhang D, Li F, Ma X. Simultaneous laparoscopic distal gastrectomy (uncut Roux-en-Y anastomosis), right hemi-colectomy and radical rectectomy (Dixon) in a synchronous triple primary stomach, colon and rectal cancers patient. *J Vis Surg.* 2016;2:101.

23. Mendez LE, Atlass J. Triple synchronous primary malignancies of the colon, endometrium and kidney in a patient with Lynch syndrome treated via minimally invasive techniques. *Gynecol Oncol Rep.* 2016;17:29-32.

24. Okoshi K, Mizumoto M, Kinoshita K. Laparoscopic subtotal proctocolectomy for synchronous triple colorectal cancers: a case report. *Asian J Endosc Surg.* 2016;9(4):303-306.

25. Rafael OC, Lazzaro R, Hasanovic A. Molecular Testing in Multiple Synchronous Lung Adenocarcinomas: Case Report and Literature Review. *Int J Surg Pathol.* 2016;24(1):43-46.

26. Yoshikawa M, Ikemoto T, Morine Y, et al. Aggressive resection of metachronous triple biliary cancer. *J Med Invest.* 2017;64(3.4):299-304.

27. Choi KW, Joo M, Kim HS, Lee WY. Synchronous triple occurrence of MALT lymphoma, schwannoma, and adenocarcinoma of the stomach. *World J Gastroenterol.* 2017;23(22):4127-4131.

28. Han Y, Shao N, Xi X, Hao X. Use of microwave ablation in the treatment of patients with multiple primary malignant tumors. *Thorac Cancer.* 2017;8(4):365-371.

29. Elec FI, Zaharie A, Ene BM, Ghervan L. Quadruple primary urogenital cancers - A case report. *Int J Surg Case Rep.* 2017;39:239-244.

30. Yeh YS, Chen CF, Lin PC, Lin CL, Huang TF, Su CM. Synchronous Appendiceal Triple Primary Neoplasms and Acute Abdomen-A Case Report. *J Acute Med.* 2018;8(4):182-185.

31. Haratake N, Takenoyama M, Edagawa M, et al. A case of different EGFR mutations in surgically resected synchronous triple lung cancer. *J Thorac Dis.* 2018;10(4):E255-E259.

32. Petroianu A, Sabino KR, Nunes MB. Synchronous triple squamous cell carcinoma of the esophagus. *Int J Surg Case Rep.* 2018;49:34-36.

33. Song L, Li Q, Yang K, Yin R, Wang D. Three primary synchronous malignancies of the uterus, cervix, and fallopian tube: A case report. *Medicine (Baltimore).* 2018;97(24):e11107.

34. Arriola AGP, Taylor BL, Ma S, Malkowicz SB, Lal P. Malignant Mixed Epithelial and Stromal Tumor of the Kidney With 2 Simultaneous Renal Carcinomas in a Male Patient: Case Report and Review of the Literature. *Int J Surg Pathol.* 2018;26(1):56-63.

35. Huang Q, He X, Qin H, Fan X, Xie M, Lian L. Triple primary malignancies in a patient with colorectal adenocarcinoma: A case report. *Int J Surg Case Rep.* 2018;42:34-37.

36. Peng L, Zeng Z, Teng X, et al. Genomic profiling of synchronous triple primary tumors of the lung, thyroid and kidney in a young female patient: A case report. *Oncol Lett.* 2018;16(5):6089-6094.

37. Zhang Y, Ge Y, Wu X, Liu S. Clinical treatment of advanced synchronous triple primary malignancies: comprehensive treatment based on targeted therapy. *Onco Targets Ther.* 2019;12:2421-2430.

38. Ying X, Zhang H, Chen B, et al. Multiple metachronous rare primary malignant tumors: A case report. *Thorac Cancer.* 2019;10(10):2050-2053.

39. Muto Y, Suzuki K, Kato T, et al. Multiple primary malignancies of six organs in a Japanese male patient: A case report. *Mol Clin Oncol.* 2019;10(5):511-515.

40. Peng C, Li Z, Gao H, et al. Synchronous primary sigmoid colon cancer and primary thyroid cancer followed by a malignant tumor of the kidney: Case report of multiple primary cancer and review of the literature. *Oncol Lett.* 2019;17(2):2479-2484.

41. Jayarajah U, Basnayake O, Wijerathne P, Jayasinghe J, Samarasekera DN, Seneviratne S. A Rare Occurrence of Three Primary Malignancies of the Rectum, Breast, and Kidney in the Same Patient: A Case Report and Review of the Literature. *Case Rep Surg.* 2019;2019:1716029.

42. Wang DD, Yang Q. Synchronous quadruple primary malignancies of the cervix, endometrium, ovary, and stomach in a single patient: A case report and review of literature. *World J Clin Cases.* 2019;7(20):3364-3371.

43. Tangsirapat V, Wongta K, Chakrapan Na Ayudhya K, Chakrapan Na Ayudhya V, Sookpotarom P. Pancreatic neuroendocrine tumor, lymphoma, and squamous cell carcinoma of hypopharynx; A case report of three primary cancers in one patient. *Int J Surg Case Rep.* 2019;65:111-114.

44. Li G, Yao J, Wu T, et al. Triple metachronous primary cancer of uterus, colon, and breast cancer: A case report and review of the literature. *Medicine (Baltimore).* 2020;99(34):e21764.

45. Te M, 3rd, Lumanlan-Mosqueda DB, Demegillo KJ. Triple Synchronous Tumors Presenting as Right Nasolabial Basal Cell Carcinoma, Papillary Thyroid Carcinoma and Prolactinoma: A Rare Case Report. *J ASEAN Fed Endocr Soc.* 2020;35(2):200-209.

46. Bai J, Xie Z, Sun L. Case Report: Metachronous Quadruple Cancers Including Breast Cancer and Triple Genital Cancer. *Int J Gen Med.* 2020;13:1575-1580.

47. AlBaqmi KH, AlMudaiheem FA, Boghdadly S, AlHussaini KA, Shokor N, AlOudah N. Multiple Primary Malignancies of the Colon, Stomach, and Kidney in a Patient with Bowel Obstruction Requiring Emergency Surgery: A Case Report. *Am J Case Rep.* 2020;21:e926472.

48. Li D, Yu M, Zhou P, Yang J, Wang Y. Whole-exome sequencing in a patient with synchronous triple primary malignancies involving lung cancer: a case report. *Precis Clin Med.* 2020;3(4):306-310.

49. Jamal E, El-Ashwah S, Ebrahim MA, et al. Case report; meta-synchronous triple malignancy in primary diagnosed CML patient. *Am J Blood Res.* 2020;10(2):22-25.

50. Maruyama N, Okubo Y, Umikawa M, et al. Quadruple Multiple Primary Malignancies: Early Detection of Second Primary Malignancy by Esophagogastroduodenoscopy/Colonoscopy Is Crucial for Patients with Classic Kaposi's Sarcoma. *Diagnostics (Basel).* 2020;10(4).

51. Zhan X, He L, Song K, Cao S, Meng E, Wang Y. Case Report: Triple Primary Malignant Tumors of the Esophagus, Stomach, and Colon in a Patient With Genetic Analysis. *Front Genet.* 2021;12:676497.

52. Aloraini AM, Helmi HA, Aljomah NA, Zubaidi AM. Multiple primary gastrointestinal tumors of gastric, pancreatic and rectal origin; a case report. *Int J Surg Case Rep.* 2021;89:106610.

53. Sumransub N, Murugan P, Marette S, Clohisy DR, Skubitz KM. Multiple malignant tumors in a patient with familial chordoma, a case report. *BMC Med Genomics.* 2021;14(1):213.

54. Abdeen Y, Al-Amer M, Taft E, Al-Halawani M. Four synchronous primary tumors in a male patient. *J Cancer Res Ther.* 2021;17(1):258-261.

55. Li Y, Ye LS, Hu B. Synchronous multiple primary malignancies of the esophagus, stomach, and jejunum: A case report. *World J Clin Cases.* 2021;9(32):9889-9895.

56. He F, Xia Y, Ling X. Diagnosis and Individualized Treatment of Three Primary Malignant Tumors: A Case Report. *Breast Cancer (Dove Med Press).* 2021;13:519-527.

57. Huang R, Li Z, Weng S, Wu S. Simultaneous triple primary malignancies, including bladder cancer, lymphoma, and lung cancer, in an elderly male: A case report. *Open Life Sci.* 2022;17(1):1263-1268.

58. Qiu B, Lin C, Wu L, Li Y. A case report of synchronous triple primary malignancies: Diffuse large B-cell lymphoma, rectal adenocarcinoma and hepatocellular carcinoma. *Front Oncol.* 2022;12:1046878.

59. Esmati E, Rezaei S, Jafari F. A case of multiple primary malignancies including peritoneal mesothelioma surviving over 11 years. *Clin Case Rep.* 2021;9(5):e04096.

60. Raikar M, Mandal S, Manas F, Kolade VO. A Rare Case of Metachronous Quadruple Primary Malignancies in a Single Patient: A Case Report and Comprehensive Literature Review. *Cureus.* 2022;14(5):e25405.

61. Du Y, Duan Y, Zhang L, et al. A Female With Synchronous Multiple Primary Malignant Tumors in the Esophagogastric Junction, Duodenum and Pancreas: Case Report and Review of the Literature. *Front Oncol.* 2022;12:890587.

62. Li ZK, Zhao Q, Li NF, et al. Synchronous triple primary malignant tumours in the bladder, prostate, and lung harbouring TP53 and MEK1 mutations accompanied with severe cardiovascular diseases: A case report. *Open Med (Wars).* 2022;17(1):2046-2051.

63. Jiang W, Zhang G, Li H, et al. Synchronous triple primary gastrointestinal malignant tumors treated with laparoscopic surgery: A case report. *Open Med (Wars).* 2023;18(1):20230742.

64. Shen H, Lv C. Multiple primary trans-systemic triple carcinoma: A rare case report. *Asian J Surg.* 2023;46(6):2466-2467.

65. Perry KW, Yankelevich G, Ashton L, Diorio G. Triple Synchronous Urogenital Malignancies of the Bladder, Kidney, and Prostate: Management in a Single Operation. *Cureus.* 2023;15(10):e47107.

66. Liu Z, Jin C, Zhang Y, Jiang Y, Wang J, Zheng L. Identification of BRAF, CCND1, and MYC mutations in a patient with multiple primary malignant tumors: a case report and review of the literature. *World J Surg Oncol.* 2023;21(1):158.

67. Cao Y. A case of rare metachronous four primary carcinoma. *J Cancer Res Clin Oncol.* 2023;149(10):7471-7477.

68. Ma XY, Tian K, Sun PF. Multiple primary malignant neoplasm: Case report and comprehensive literature review. *Front Oncol.* 2022;12:1090634.

69. Nguyen DT, Nguyen LM, Phan TL, Bui QV. One Patient With 4 Different Primary Cancers: A Case Report. *Clin Med Insights Case Rep.* 2023;16:11795476221150597.

70. Park CL, Moria F, Saleh RR. Combination of Osimertinib with Concurrent Chemotherapy and Hormonal Therapy for Synchronous NSCLC, Hormone Receptor-Positive Breast Cancer, and Triple-Negative Breast Cancer: Case Report. *Case Rep Oncol.* 2023;16(1):1080-1086.

71. Chen Y, Luo S, Zheng Q, et al. A case of metachronous triple primary carcinoma complicated with pulmonary tuberculosis: Case report and review. *Medicine (Baltimore).* 2024;103(38):e39638.

72. Tran LMB, Do MD, Nguyen TH, Vuong NM. Rare occurrence of triple primary malignant tumors: Dermatofibrosarcoma protuberans, lung adenocarcinoma and papillary thyroid carcinoma in a patient with genetic evaluation. *Radiol Case Rep.* 2025;20(2):1194-1199.

73. See XY, Omer A, Tang Z, Eid F, Zambon M. Metachronous and Synchronous Triple Primary Lung Cancers in a Chronic Smoker. *J Community Hosp Intern Med Perspect.* 2024;14(2):100-103.

74. Liu Y, Yang H, Fu X, et al. BRCA2, PALB2, RECQL4 Germline Pathogenic Variants, and Somatic TP53 Mutation in Triple Metachronous Malignancies: A Case Report and Literature Review. *Int Med Case Rep J.* 2024;17:23-29.

75. Wu H, Jiang Y, He M, Xu X, Jiang H. Multiple primary tumors in a patient with non‑small‑cell lung cancer harboring mutations in ERCC6 and LYL1: A case report. *Oncol Lett.* 2025;29(1):63.

76. Hong Z, Tongsong Z, Cunhai C, Xiao L, Haiping S. Case report of multiple primary cancers and results of genetic testing to preliminarily explore their pathogenesis. *SAGE Open Med Case Rep.* 2024;12:2050313X241252371.

77. Porav-Hodade D, Gherasim R, Loghin A, et al. Bladder Adenocarcinoma in a Constellation of Multiple Site Malignancies: An Unusual Case and Systematic Review. *Diagnostics (Basel).* 2024;14(22).

78. Seyyedi MS, Zangouri V, Dehghani Z, Dehghanian A, Jahromi MG. A rare occurrence of breast, thyroid, and stomach tumors in a single patient: A case report. *Int J Surg Case Rep.* 2024;119:109670.

79. Bi XR, Zhao SY, Ma YQ, et al. Multiple primary cancers with gastrointestinal malignant tumors as the first manifestation: Three case reports and review of literature. *World J Gastroenterol.* 2025;31(8):100146.
